# Role of Antisperm Antibodies in Infertility, Pregnancy, and Potential for Contraceptive and Antifertility Vaccine Designs: Research Progress and Pioneering Vision

**DOI:** 10.3390/vaccines7030116

**Published:** 2019-09-16

**Authors:** Vickram A. S., Kuldeep Dhama, Sandip Chakraborty, Hari Abdul Samad, Shyma K. Latheef, Khan Sharun, Sandip Kumar Khurana, Archana K., Ruchi Tiwari, Prakash Bhatt, Vyshali K., Wanpen Chaicumpa

**Affiliations:** 1 Department of Biotechnology, Saveetha School of Engineering, Young Scientist DST-SERB, Govt. of India, Saveetha Institute of Technical and Medical Sciences, Chennai 600077, Tamil Nadu, India; 2 Division of Pathology, ICAR-Indian Veterinary Research Institute, Izatnagar, Bareilly 243122, Uttar Pradesh, India; shyma05vet@gmail.com; 3 Department of Veterinary Microbiology, College of Veterinary Sciences and Animal Husbandry, R.K. Nagar, West Tripura 799008, India; chakrabortysandip22@gmail.com; 4 Division of Physiology and Climatology, ICAR-Indian Veterinary Research Institute, Izatnagar, Bareilly 243122, Uttar Pradesh, India; hariabdulsamad@gmail.com; 5 Division of Surgery, ICAR-Indian Veterinary Research Institute, Izatnagar, Bareilly 243122, Uttar Pradesh, India; sharunkhansk@gmail.com; 6 ICAR-Central Institute for Research on Buffaloes, Sirsa Road, Hisar 125001, Haryana, India; sandipkk2003@yahoo.co.in; 7 Department of Biotechnology, School of Biosciences and Technology, Vellore Institute of Technology, Vellore 632014, Tamil Nadu, India; archanatnau@gmail.com (A.K.); vyshali93@gmail.com (V.K.); 8 Department of Veterinary Microbiology and Immunology, College of Veterinary Sciences, Deen Dayal Upadhayay Pashu Chikitsa Vigyan Vishwavidyalay Evum Go-Anusandhan Sansthan (DUVASU), Mathura 281001, India; ruchi.vet@gmail.com; 9 Teaching Veterinary Clinical Complex, College of Veterinary and Animal Sciences, Govind Ballabh Pant University of Agriculture and Technology, Pantnagar 263145 (Udham Singh Nagar), Uttarakhand, India; drbhatt_p@rediffmail.com; 10 Center of Research Excellence on Therapeutic Proteinsand Antibody Engineering, Department of Parasitology, Faculty of Medicine Siriraj Hospital, Mahidol University, Bangkok 10700, Thailand

**Keywords:** antisperm antibodies, infertility, pregnancy, sperm antigens, contraception, antifertility vaccine

## Abstract

Sperm of humans, non-human primates, and other mammalian subjects is considered to be antigenic. The effect of changes in autoimmunity on reproductive cells such as spermatozoa and oocytes play a critical but indistinct role in fertility. Antisperm antibodies (ASAs) are invariably present in both females and males. However, the degree of ASA occurrence may vary according to individual and gender. Although the extent of infertility due to ASAs alone is yet to be determined, it has been found in almost 9–12% of patients who are infertile due to different causes. Postcoital presence of spermatozoa in the reproductive tract of women is not a contributory factor in ASA generation. However, ASA generation may be induced by trauma to the vaginal mucosa, or by anal or oral sex resulting in the deposition of sperm inside the digestive tract. It is strongly believed that, in humans and other species, at least some antibodies may bind to sperm antigens, causing infertility. This form of infertility is termed as immunological infertility, which may be accompanied by impairment of fertility, even in individuals with normozoospermia. Researchers target ASAs for two major reasons: (i) to elucidate the association between ASAs and infertility, the reason ASAs causes infertility, and the mechanism underlying ASA-mediated infertility; and (ii) to assess the potential of ASAs as a contraceptive in humans in case ASAs influences infertility. Therefore, this review explores the potential application of ASAs in the development of anti-spermatozoa vaccines for contraceptive purposes. The usefulness of ASAs for diagnosing obstructive azoospermia, salpingitis, and oligoasthenoteratozoospermia has been reviewed extensively. Important patents pertaining to potential candidates for spermatozoa-derived vaccines that may be utilized as contraceptives are discussed in depth. Antifertility vaccines, as well as treatments for ASA-related infertility, are also highlighted. This review will address many unresolved issues regarding mechanisms involving ASAs in the diagnosis, as well as prognoses, of male infertility. More documented scientific reports are cited to support the mechanisms underlying the potential role of ASA in infertility. The usefulness of sperm antigens or ASAs (recombinant) in human and wild or captive animal contraceptive vaccines has been revealed through research but is yet to be validated via clinical testing.

## 1. Introduction

Sperm has been considered as being antigenic toward the female body for a long time [1]. Various sperm-associated proteins causing antigenicity have been identified and characterized, and their role in fertility disorders has been described. Antisperm antibodies (ASAs) are naturally formed against such antigenic proteins following the exposure of these proteins to immune cells. Many studies are being conducted on this subject with a multifactorial approach. A disruption must occur in the blood–testis barrier (BTB) for ASAs to be generated, and in humans, ASAs have been associated with 2–50% of infertility incidences [2,3]. Changes in the masculine reproductive tract, which is substantially made up of smooth muscles and immune components, have been distinctly investigated, but not in the context of infertility and its associated problems. Recent studies pertaining to ASA-associated infertility conditions have unraveled the interaction between these two systems, suggesting a potent role of ASAs in the infliction of immunological infertility [4,5].

ASAs were believed to be present in both males and females in considerable amounts [6]. ASAs were found in higher concentrations (approximately 9–12%) among infertile patients [3]. Reportedly, ASAs are also present in fertile men and fertile women for unknown reasons or the function is still not explored properly [7]. This fact indicated that the mere presence of ASAs does not directly disrupt fertility or the fertilization process. The presence of ASAs in infertile men was first reported by Rumke and Wilson in 1954 [8,9]. Since then (particularly during the period between 1960 and 2000), researchers have focused their attention on ASAs. More recently, research has been focused on the use of ASAs as a contraceptive [10,11]. For human males, risk factors associated with ASA formation include the blood–testis barrier breakdown, surgical trauma, various microbial infections, prostitis and orchitis, testicular cancer, varicocele, and unsafe sex (either anal or oral sex). Various autoimmune studies confirmed that molecular mimicry toward bacteria like *Escherichia coli* can also induce ASA-mediated male infertility [12]. However, the cause of the inconsistencies associated with the generation of ASAs in females, where some generate ASAs while others do not, remain unclear. Furthermore, women whose male partners carry ASAs in their semen, usually carry ASAs, but such ASAs react only with the sperm of the partner and not with the sperm of other males. In the case of women, the presence of spermatozoa in the reproductive tract following intercourse is not a contributing factor for the genesis of ASAs. However, there may be a probability for ASA generation during intercourse if there is trauma to the vaginal mucous membrane or if sperm is deposited in the digestive tract due to anal or oral sex [13,14,15]. Higher levels of ASAs were detected in patients who were clinically positive for testicular carcinoma [16], testicular torsion [17], epididymal and bilateral orchitis [18], varicocele [19], seminal infections [20], sexually transmitted diseases [21], prostate inflammation [22], inflammation in seminal vesicles, infections in male reproductive tract [20], destruction in seminiferous tubules [23], vasectomy, vasectomy reversal, ejaculatory dysfunction, and erectile dysfunction [24].

Reportedly, human seminal ASAs exert undesirable effects on sperm quality by altering major semen parameters. Numerous contradictory scientific documents exist; some of them contend that ASAs affect major semen parameters as indicated by statistically significant differences in the semen parameters between ASA groups and fertile groups without ASAs [25], while others suggest that there is no statistical difference in the semen parameters between ASA men and normal men, and that ASA presence seems to be innocuous for fertility. In a comparative study for assessing male infertility factors associated with infection, inflammation, and autoimmunity, it was reported that murine models exhibit the presence of low titers of ASAs in their sera, even 6–7 months after a vasectomy [26]. Serum ASAs can be used as a diagnostic tool to identify obstructive and non-obstructive azoospermia [27]. It is interesting to note that regarding fertilization among human groups with various ASA levels, there were no differences in the velocity and rates of cleavage, proportion of good quality embryos, pregnancies (clinical), or miscarriages [28]. Active spermatogenesis plays a key role in ASA detection for apt diagnosis of whether the problem lies with sperm production or transport, or during storage or ejaculation [29]. Many hypotheses related to ASA-mediated infertility have been put forward where a few have proposed that implantation was affected, whereas others have proposed that embryo development was involved. According to a report published by the World Health Organization (WHO), any autoimmune response against sperm cells was proved to be a cause for male infertility by itself.

The sperm antigenicity has been known for centuries. Infertility may develop in humans as well as other species due to the binding of certain antibodies to the surface of sperms. Interest in these antibodies has increased in recent years due to the following two reasons: (i) detection of antibodies associated with infertility is required to treat infertile couples properly and accurately; and (ii) because such antibodies induce infertility, these antibodies may be developed for contraception purposes, as well as for regulating feral animal populations [30].

Sperm cells entering the lower female reproductive tract are considered to be allogeneic antigens by the female body [31]. Under these circumstances these allo-antigens can initiate allo-immunity responses in the female body. These allo-antigens expose the female immune system to pathologic conditions, resulting in a unique inflammatory or allergic reaction, which leads to the production of antisperm antibodies (ASAs) [32,33]. Many studies have demonstrated that ASAs may inhibit sperm motility [29] and sperm progression [34], in addition to initiating differences in sperm function and reducing the fertilizing capacity of sperm [35].

Studies have shown seminal ASAs, as well as ASAs circulating in serum, affect sperm production, sperm transport, implantation, early embryonic development, gamete interaction, and fetal development [7]. Some researchers reported that there was no association between seminal ASA levels, pregnancy rates, and Intra cytoplasmic sperm injection/*In vitro* fertilization (ICSI/IVF) pregnancies. In general, male contraception may be implemented using any one of the following [36]:preventing the rendezvous and union of male and female gametes (sperm and ovum) via the use of physical barriers, such as condoms during intercourse; vasectomy; experimental vas occlusion methods, such as experimental epididymal knots; disturbing sperm production and transport via experimental hormonal and non-hormonal methods;and by killing or inhibiting sperm from encountering an ovum following ejaculation via spermicides and other antibodies [36,37]. Many researchers have postulated that ASAs may be useful for producing antifertility vaccines for contraception. Antisperm antibodies that are considered to be a candidate for vaccines must be more effective than existing contraceptives in their contraceptive properties and be reversible [38]. The first study conducted by Baskin in 1932 suggested that ASAs may be the best contraceptive due to the absence of any harmful effects to men and women. The most successful antigen isolated was hyaluronidase (PH-20), which completely abolished fertility in male guinea pigs with minor side effects [39]. The current review focuses on the effect of ASAs on obstructive azoospermia, oligoasthenoteratzoospermia, salpingitis, various semen parameters, sperm transport, sperm acrosomal reaction, and complementary actions. This review also analyzes the role of ASAs in designing successful antispermatozoa vaccines for the purpose of male and female contraception. The most successful patents in the field of contraception for both males and females are also discussed.

## 2. Role of Antisperm Antibodies in Infertility

### 2.1. Detection Methods for Antisperm Antibodies

Antisperm antibodies can be detected in both male and female subjects through specific immunological reactions [40]. Ina female patient’s serum, sperm immobilizing antibodies are detected through an immobilization test. In the case of males, ASAs on the sperm surface are detected directly through a proper immunobead test or through a mixed antiglobulin reaction test [41,42].

Clarke et al. studied the specificity and reproducibility of the immunobead test (IBT) for the detection of ASAs in 183 samples. The comparison of IBT and SIT (sperm immobilization test) in the serum samples and sperm agglutination in semen samples concluded that IBT is an immunologically specific test with a 97.5% reproducibility. A positive IBT group signifies the incidence of sperm agglutination at a significant level. This study suggests that IBT is the most accurate test for the routine screening of men with ASA [43].

Computer assisted semen analysis (CASA) is used for detecting the presence of ASAs in the semen and serum by using an optimized custom-designed program [44,45].

Dondero et al., studied the correlation between the direct immunobead test with other antisperm antibody detection methods. The gelatin agglutination test (GAT) and tray agglutination test (TAT) were used as indirect methods to detect the ASA and the direct IgG mixed antiglobulin reaction test (MAR) is a direct method to detect the sperm antibodies bound on to the surface of the sperm. These results showed a better positive correlation between the direct and indirect methods and helped to design a mathematical prediction model to establish a good regression [46,47]. Lenzi et al. also studied the correlation between direct and indirect methods. The result also enables the application of a mathematical model to any biological model that could explain the antibody test. This study led to set of predictive thresholds to explain the significance of direct and indirect methods [48].

### 2.2. Diagnosis of Obstructive Azoospermia UsingSerum ASA Values

Three major conditions that need to be diagnosed in infertile men are sperm production (active spermatogenesis), sperm transport until maturation, and ejaculatory functions [24]. Spermatogenesis is the major step via which sperm is produced, wherein innumerable active antigens on the surface of sperm are capable of instigating immune responses in the female body. Under these circumstances, any disturbance in the blood–testis barrier (BTB) will directly lead to ASA production [49]. Major causes for higher levels of ASA production are a vasectomy and the congenital absence of bilateral or unilateral vas deferens [50], while some minor causes for ASA production are acute or chronic epididymitis [51], trauma to the testes [52]_,_ and cryptorchidism [53]. Detection of active spermatogenesis is a prerequisite for the diagnosis of fertile and infertile men [54]. Therefore, evaluating ASA production may enable issues with sperm production, sperm transport, or sperm ejaculation to be identified if they are present [55]. While ASAs were detected in 88% of the vasectomized males, only a small proportion of vasectomized males (8%) were found to be ASA-positive, as reported by Marconi and Weidner [55]. The sensitivity of serum ASAs in the diagnosis of obstructive azoospermia was found to be 90%, with a specificity of 93% in this study, which used infertile patients (nonazoospermic) as a control group.

The combined sensitivity, as well as specificity, of various subtypes of ASAs, viz., IgG, IgM, and IgA, were not found to be higher than all ASAs taken together. Serum ASAs enhance the accuracy of an obstructive azoospermia diagnosis. This also ensures the existence of active testicular spermatogenesis [56]. The permanent association of chronic obstruction of the male reproductive tract with ASA production is also evident from such studies [57]. Lee et al. evaluated serum ASAs as a diagnostic marker to predict the obstruction of the vas deferens and epididymis. This can also be used to correctly diagnose obstructive azoospermia, and is a major predictor or marker of obstructive and non-obstructive azoospermiaas well [27]. Lee et al. studied 484 patients, out of which, 270 exhibited obstruction in either the vas deferens or epididymis. All these men had undergone a vasectomy. Nearly 88% of those presenting with obstruction displayed increased levels of serum ASAs. By contrast, only 8% of those without any documented obstruction exhibited serum ASAs. In patients with a varicocele, nearly 44% possessed serum ASAs. Serum levels of IgG, IgA, IgM, and serum ASAs were analyzed for their potential as diagnostic markers of obstruction-related problems. ASA levels in the obstructed group were significantly higher than those of the non-obstructed group. Serum IgG levels in the varicocele group were higher when compared with other subgroups. Thus, it may be concluded that the evaluation of serum ASA levels in infertile human groups is a non-invasive and comparatively cheaper technique for detecting obstructive azoospermia from other conditions associated with infertility [27].

### 2.3. ASA and Salpingitis

Over 10–15% of females encounter issues of infertility in varying degrees during their reproductive age [58]. Female infertility has been considered a significant issue in both developing and developed countries for decades [59,60]. Infertility in women could be attributed to different etiologic factors such as endometriosis, conditions related to ovulation, menstruation associated issues, tubal factor diseases, fallopian tube blockages, and uterine abnormalities [61]. Of these, tubal factors are considered to be the major cause underlying female infertility [62]. Among tubal factors, pelvic inflammation and salpingitis are considered to be the major reasons for infertility [63,64,65]. Salpingitis is related to the instigation of an enhanced immune response and actively participates in antibody induction [66]. Although serum ASA definitely reduces the capacity for fertilization in females, many studies have not investigated the correlation between ASA levels and chronic salpingitis. Recently, Liu et al. investigated the correlation between serum ASA levels and salpingitis-associated female infertility [67]. When female reproductive tracts suffer an increasing infection involving the cervix and the vagina initially, salpingitis is indicated [65,68]. The mechanism underlying ASA generation during fallopian tube inflammation is still unclear. An elevation of sperm immunity levels is considered to be the factor responsible for this phenomenon. Elevation of ASAs in the female genital tract is both local and systemic [69,70].

Microbes are transmitted sexually, viz., *Chlamydia trachomatis*. The human immunodeficiency virus (HIV) is responsible for causing inflammation and for ultimately infecting the genital tract in females [71,72]. In due course, genital tract epithelia become defective, and such impairment may lead to the loss of immune system integrity involved in cellular and humoral defense mechanisms. Eventually, the interaction between sperm antigens and cells of the immune system in the genital tract is augmented. This enhancement of the immune response instigated by sperm antigens results in a gradual generation of ASAs [67]. Of the 48 female patients diagnosed with chronic salpingitis, 28 had high serum ASA levels. Bound ASA-IgA was detected in these 28 patients using an indirect immunobead assay, thereby confirming a correlation between ASA levels and salpingitis-associated female infertility. The fallopian tubes are considered to be an important organ in the female reproductive system, following the ovary and the uterus. Sperm acrosome reactions, sperm and zona binding at the ampullary-isthmic junction, very early embryonic development, and further oocyte-sperm interactions occur only in fallopian tubes [72]. Any abnormalities found in the fallopian tubes results in tubal-factor female infertility. During salpingitis, genital tract infections are found to be an important factor causing inflammation in the fallopian tubes [72]. The mechanism underlying ASA generation during salpingitis remains unclear [50]. ASAs of local and systemic origins were found in the female reproductive tract [67,69]. In this study, Liu concluded that patients with chronic salpingitis exhibited higher ASA levels, and that immune factors also need to be focused on for the effective treatment of salpingitis [67].

### 2.4. ASA and Oligoasthenoteratozoospermia (OAT)

Oligoasthenoteratozoospermia (OAT) is considered to be a severe abnormality associated with the quality and quantity (volume) of semen [73]. Men with severe OAT have a lower number of sperm cells in their ejaculate, decreased mass motility, less progressive forward motility, and a lower number of sperm with normal morphology [74]. These abnormal seminal parameters alter the functions of the testes and the epididymis. Infertile men with severe OAT exhibit complicated etiologies and are considered to be a rare infertile group. Researchers believe that idiopathic OAT may be associated with an activated immune system, leading to ASA production [75]. Semen samples of OAT patients display increased expression levels of ASA and bind with actin-binding proteins. Studies have analyzed the role of ASAs in the pathogenesis of olioasthenoteratozoospermia [76]. Xu et al. investigated the correlation between serum ASA levels and the pathogenesis of OAT in OAT patients. Male infertility is often associated with ASAs, particularly in the case of OAT men [75]. A variety of etiologies have been predicted for the most frequent OAT phenotypes, including alterations in post-testicular and testicular organs, non-inflammatory functions, age, gamete alterations, environmental pollutants, and mitochondrial alterations [77]. The most common etiology of OAT was varicocele, oxidative stress, and hormonal imbalance, leading to poor semen quality [77]. Xu et al. analyzed the ASA levels of more than 50 OAT patients. Only two or three OAT patients were positive for ASAs, while the rest did not induce any form of immune response to produce ASAs [75]. Xu et al. concluded that OAT was not associated with higher levels of ASAs, but that evaluation of ASAs in the OAT group was still quite normal, despite no correlation of ASAs with OAT pathogenesis [75,78].

### 2.5. ASA and Normospermia and Vasectomy Reversal

Estimates suggest that over 85–90% of pregnancies are viable for healthy couples, whereas only 40–45% are viable in the case of vasectomy reversal cases [79]. The major difference is the presence of ASAs in vasectomy-reversal couples [80]. ASAs kill several active sperm and at times make sperm cells immotile, leading to necrozoospermia [81]. Mechanisms underlying the prevention of conception or pregnancy by ASAs are poorly understood and sufficient data are not available for debate. Necrospermia itself is poorly documented [82], even though it is estimated that 0.2–0.4% of infertility is due to necrospermia [83]. Necrospermia is indicated in many etiologies, including chronic or acute medical conditions, infections [84], severe exposure to pollutants [85], and epididymal necrospermia [86], among others. Sperm degeneration and death may occur during the epididymal passage or storage time, or because of biochemical components present in seminal plasma during ejaculation [87]. Chavez–Badiola et al. attempted to successfully retrieve active sperm from necrospermic patients with ASAs following vasectomy reversal procedures [87]. ASAs are said to be produced locally in the epididymis. Thus, testicular retrieval of sperm [88], before the sperm reached the epididymis and mixed with seminal plasma, was considered to be beneficial for necrospermia patients with a vasectomy reversal [87].

### 2.6. ASA in Cryptorchidism

Cryptorchidism is a major genital abnormality that affects 0.5 to 3% of one- to two-year-old boys [89]. If untreated, it may lead to infertility in adulthood, especially in those with bilateral undescended testes. Normal spermatogenesis was found to occur in the case of unilateral cryptorchidism, in which case, some other additional deferential factors causing infertility needs to be focused on [90]. An orchiopexy is a state-of-the-art treatment commonly used in 2–3-year-old boys to prevent adulthood infertility, but it is not evident whether treating boys at very early stages will lead to changes in semen parameters or alterations in immune responses leading to change in testis function at older or reproductive ages [91]. Infertile patients with cryptorchidism exhibit an increased incidence of ASAs. Many reports are also available showing that even before attaining puberty, boys were found with serum ASA levels and abnormalities in the genital tract [91]. Mechanisms underlying the occurrence of serum ASA levels in cryptorchidic boys are still not well understood, even after various studies [92,93,94]. In order to explore the role played by ASA in the inhibition of future fertility in cryptorchidic boys, 32 boys who had undergone an orchiopexy and 21 boys who had undergone inguinal surgeries of different kinds were examined by Miniberg et al. in 1993 [95]. The purpose of the study was to detect the presence of antibodies to donor sperm. ASAs were detected in 28% of the boys via immunobead assays (indirect) and such ASAs were mainly found to be of the isotype IgG. However, only 4% of the boys were found to be positive for ASAs in the comparison group [94]. Sinisi et al. studied a larger population, which included unilateral and bilateral cryptorchidic subjects and analyzed their ASA levels to determine the presence of a correlation between pubertal changes, age, and surgery [96]. This study concluded that although ASAs were present in all the cryptorchidic boys who participated, the presence of ASAs was independent of all other parameters, such as testicular location, pubertal changes, and age [77].

### 2.7. ASA Influence on Semen Parameter

Cui et al. [7] studied the influence of ASAs on different semen parameters using a systematic meta-analysis [7], where the effect of ASAs was analyzed using a random-effects model. The model allowed the effect of each semen parameter, such as sperm counts, viability, sperm normal morphology, sperm motility, and the influence of ASAs, to be analyzed [7]. Data related to sperm liquefaction and total volume of semen during ejaculation were analyzed through a fixed-effects model. Cui et al. found a significant difference in sperm count and sperm motility between the ASA-positive patient group and the ASA-negative group [7,97]. The study did not indicate a significant difference between other semen parameters of ASA-positive and ASA-negative groups, including morphology and volume of ejaculation. In the fixed-effects model, semen liquefaction time showed a significant difference in the ASA-positive group with elapsed time, whereas it was normal for the ASA-negative group [98]. Sub-meta-analysis was performed only for sperm counts and sperm motility in the random-effects model. This analysis indicated a significant difference between ASA-positive and ASA-negative groups, with lower levels in the ASA-positive group [7,98]. Overall, these results suggest that the presence of ASAs in semen significantly reduced the sperm concentration and sperm motility of the ASA-positive infertility males compared to the ASA-negative males [99,100,101,102,103,104,105,106]. The effect of ASAs on sperm morphology, sperm viability, semen volume, sperm progressive motility, and sperm abnormal morphology was not identified in all the studies conducted.

### 2.8. VaricocelesAssociated with ASAs

A varicocele, which is marked by pathological engorgement of spermatic veins, is present in approximately 11.7% of men with normal values during semen analysis, while it is prevalent in approximately 25.4% of men who display certain aberrations from the normal range with respect to different semen parameters [107]. This clinical condition is concomitant with defective testicular function occurring via numerous mechanisms, such as shifts or adaptive tuning in the hypothalamic–pituitary–gonadal (HPG) axis, venous stasis due to hypoxic insult to the germinal epithelium, loss of control and regulation with respect to the counter-current exchange system, resulting in elevated testicular temperature and backward streaming of noxious metabolites from the kidneys or the adrenal gland [108]. ASAs are an important cause of infertility in 8–21% of infertile men and adversely affect the fertility of patients with a varicocele, with a minor but reproductively significant decrease in seminal parameters such as motility and concentration [109]. ASA levels of infertile men with a varicocele were studied by Mehrsai et al. in 2005. In this experimental survey, over 30 men with a varicocele were studied during their surgical varicocelectomy [110]. All patients in this group were evaluated via a physical examination of the varicocele. Semen samples in varicocele patients were collected before and after a varicocelectomy [25,111]. These samples were evaluated for the presence of ASAs via indirect immunobead assay (IBT). Four different groups were compared: varicocele patients, varicocele patients before varicocelectomy, varicocele patients after varicocelectomy, and control groups. ASA levels were higher in the varicocele patients group when compared with other groups. ASA concentrationswerelower in the varicocelectomy group only [112]. There was a significant reduction in the quality of semen in ASA-positive varicocele patients, in parallel with the grade of spermatic cord vein dilatation. Those patients showing no improvement of spermiograms following a varicocelectomy exhibited greater levels of ASAs [112,113].

### 2.9. Circulating ASAs in Polycystic Ovarian Syndrome (PCOS)

Polycystic ovarian syndrome (PCOS) is the most common cause of infertility in females of reproductive age [114]. Most infertile women with PCOS exhibit anovulation, high testosterone levels, and obesity. In many cases, PCOS is also associated with mild inflammation, injury, and ovarian damage [115,116]. These factors result in the formation and exposure of self-antigens, leading to the formation of auto-antibodies. Some women naturally have allo-immunity against sperm cells, resulting in the production of ASAs, which also interfere with female fertility [117,118]. PCOS is an excellent example of a pathological condition that reveals multistage/complex interactions involving the immune system and various aspects of the endocrine system. In autoimmune cases, immunologically active cells are stimulated by exposure to hidden antigens on sperm surfaces [119]. Many authors reported that the presence of ASAs circulating in serum in women may influence PCOS, resulting in infertility [120,121]. ASAs, along with antiphospholipid autoantibodies, are reported in female subjects with infertility, suggesting the involvement of immunologic disturbances in causing infertility [122]. Yang et al. studied the serum circulating levels of ASAs in PCOS women and correlated PCOS-induced infertility and the effect of ASAs on the pathogenesis of PCOS [123]. Nearly 46 infertile women with PCOS participated in this study. Circulating serum ASA levels, as well as sex hormones, were measured in all the patients [124,125]. ASA levels were measured using an indirect immunobead assay (IBT). Sera of four to five patients displayed low levels of circulating ASAs, while none of the patients showed elevated levels of ASAs in their serum [28,125]. This study concluded that there was no correlation between circulating ASAs and the pathogenesis of PCOS-associated infertile females. Measuring ASAs in serum is still followed routinely to detect the etiology of female infertility in most hospitals [126].

### 2.10. ASAs and TheirInfluence on the Sperm Acrosome Reaction

ASAs can influence the fertility of men by binding to the sperm surface. Reportedly, sperm-immobilizing antibodies directly block fertilization in humans by interfering with crucial events of capacitation and the release of acrosomal enzymes, which aid spermatozoa in penetrating different layers of oocytes [127]. ASAs are found to affect sperm–ovum interactions, acrosome reactions (ARs), sperm capacitation, sperm motility, sperm density, sperm viscosity, and binding to zona pellucida [128,129,130]. Associated studies are considered to be controversial. Acrosome reactions are visualized in vitro with the help of monoclonal antibodies (mAbs) for the specific structures exposed by sperm cells [131,132]. This technique was used by researchers to differentiate between acrosome-reacted and unreacted sperm cells in an ejaculate. Binding of mAbs to active sperm cells may be visualized via fluorescein isothiocyanate (FITC) staining and one of the most effective instruments to visualize this is flow cytometry. Propidium iodide (PI) was used as a supravital staining agent for detecting vital and avital cells [133]. Bohring et al. studied the influence of ASAs on acrosome reactions by using mAbs and flow cytometry [131]. This study was designed using three groups: group 1 consisted of seminal plasma free control samples, group 2 consisted of control samples with seminal plasma and without ASAs [131], and group 3 was a test group consisting of control samples carrying seminal plasma and ASAs. Each sample was divided into two different portions in order to separately investigate induced acrosome reactions (IAR) [132,133] and spontaneous acrosome reactions (SAR) [134,135,136]. Induced acrosome reactions were conducted using calcium ionophores [135]. Fewer ARs were detected in case of sperm cells without seminal plasma group when compared with sperm cells with seminal plasma but without ASAs [132]. This study concluded that ASAs collected from patients with immunological infertility were sufficiently mature to carry the ARs forward in donor semen. Inhibition of IAR and SAR by ASAs was not evident. It was confirmed that one or two sperm antigens were required to carry the ARs forward [131]. In animal reproduction studies, ASAs were found to affect fertility, especially in bovine species. IgA antibodies bound to the bovine spermatozoa surface-inhibited variation of plasma membrane fluidity during capacitation, thereby blocking the effective binding of spermatozoa to the zona pellucida of an ovum, resulting in infertility [136].

### 2.11. Influence of ASAs on Sperm Transport

Sperm are transported within the female reproductive tract until sperm safely reach the oocyte and bind to specific receptors on the surface of its zona pellucida, resulting in fertilization; this is considered to be an important event for proper fertilization [137]. Cervical mucus acts as a filter aid for sperm travelling in the reproductive tract [138,139]. It is believed that most immunological reactions and ASA binding happens in the lower reproductive tract of the female. ASAs are believed to inhibit sperm penetration in the cervical mucus [140]. Cervical-mucus-derived ASAs will immobilize the sperm and prevent its passage through the mucus [141]. Evidence suggests that IgA-derived ASAs attach to the head of sperm migrating through the cervical mucus. Similarly, IgM- and IgG-derived ASAs attach to the sperm mid region. This will impair the ability of sperm to travel through the cervical mucus [142,143]. Mechanisms underlying ASA interference in active sperm cell transport in the cervical mucus have been extensively studied. Kremer and Jager studied the binding of glycoproteins by secretory components of IgA-derived ASAs in the cervical mucus that leads to sperm oscillations in the cervical mucus [144]. The same mechanism was not observed in IgG-derived ASAs in the cervical mucus [144]. These findings suggest that cervical mucus may act as an immunological filter that allows only the most active fertile mature sperm cells to reach the oocyte and stops all other sperm cells coated with antisperm antibodies of any type [144].

### 2.12. ASAs in the Complement System

The complement system, which is a set of reactions that finally ends up in the lysis of cells, involves a cascade of many proteins that bind to the antigenic surface of cells. Out of IgG, IgA, and IgM, IgM and IgG were found to be good activators of the complement system, whereas IgA was a poor activator [145]. D’Cruz et al. studied the mobility of sperm using incubation with IgA-derived ASAs [146]. It was found that there was a significant reduction in the mobility of sperm cells and a drastic change in the morphology of sperm cells [147]. D’Cruz et al. also studied the effect of sperm-bound ASAs isolated from infertile men, which helped in elucidating complement activation [148]. Price et al. demonstrated the presence of the complement system in the lower and upper reproductive tract, especially in the cervix/cervical mucus [149]. It was found that approximately one-tenth of the complement system was present in the cervical mucus compared to the blood complement system [149].

## 3. Role of Antisperm Antibodies in Pregnancy

Several investigations have been undertaken to elucidate the direct/indirect relationship between the presence of ASAs and the subsequent pregnancy rate in females, but the results have been inconsistent, especially when advanced assisted reproductive techniques were involved. Although, in males, ASAs were directly associated with male infertility, the overall effect of ASAs on reproductive efficiency in females is yet to be explored, even though reports indicate a reduction in spontaneous pregnancies [150,151].

### ASAs and Missed Abortions

Researchers provided evidence that antibody-mediated immune reactions were responsible for spontaneous or missed abortions [152,153]. Missed abortion is a term used for a spontaneous abortion due to fetal demise without any extraneous intervention. The causes for these missed abortions are intricate, and many immune factors play crucial roles [145]. A series of immunological reactions should occur for a successful pregnancy to take place, and the female body should be able to adapt to these reactions. Hence, a successful pregnancy does not depend on fetal–maternal interactions alone [154]. A vital question related to ASA function is: Does it play a role in missed abortions or not? [155]. Many studies have posed this question, but a proper answer was not found until the 2011 studies of Wang and Zhu [156]. Many previous reports have suggested that a woman with serum ASAs are at a higher risk for a spontaneous abortion. Many studies suggest an absence of correlation between the levels of ASAs circulating in sera and the spontaneous loss of pregnancy. Wang and Zhu tested a group of 64 spontaneous abortion patients using an immunobead assay method. Of these 64 patients, only one showed interactions with circulating ASAs [156]. This finding proved the absence of a correlation between levels of ASAs and spontaneous abortion. This result also demonstrated that women are not etiologic for increased/decreased levels of ASAs leading to a missed abortion. Statistically and clinically, ASAs have no role in missed abortion, although it causes infertility. Nevertheless, ASAs need to be routinely evaluated in missed abortion and infertile patients in order to obtain a comprehensive understanding of the role of ASAs in infertility [157,158].

**Figure 1:**
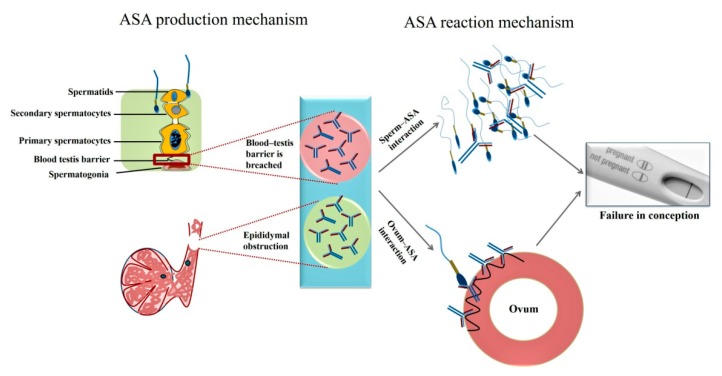
Antisperm antibody (ASA) production by a male and its interaction mechanisms with the female leads to conception failure.

An overview on ASA production and interactions with ovum is depicted in Figure 1.

**Figure 2:**
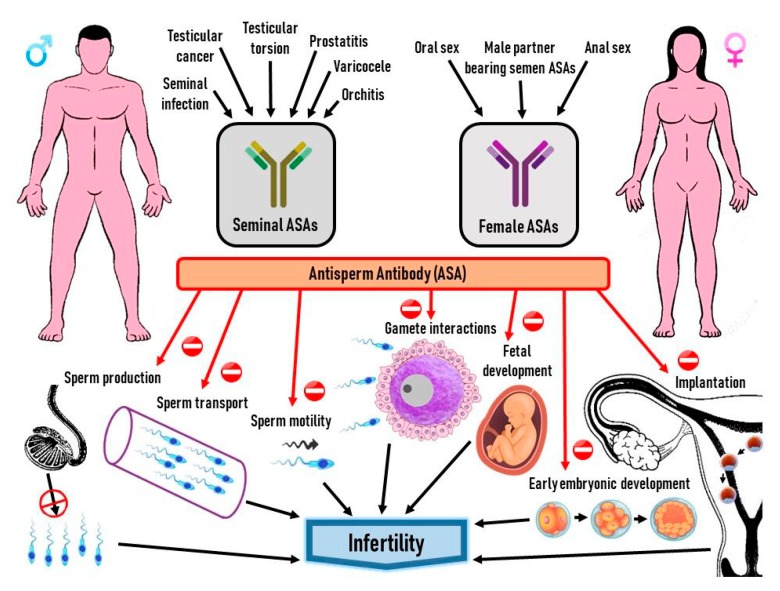
Production of antisperm antibodies and the mechanisms of action in the female reproductive tract that lead to infertility.

Production of antisperm antibodies and the mechanisms of action in the female reproductive tract that lead to fertilization failure are presented in Figure 2.

## 4. Role of ASAs in Designing Effective Contraception

### 4.1. Antifertility Vaccines

Developing antispermatozoa vaccines for males is a challenging task for researchers as the spermatozoa number is in the millions and not in the tens, hundreds, or thousands [10]. Immunology of the male reproductive system is poorly understood [159]. Antisperm vaccines for men are achieved via the induction of IgA secretion in the prostate, which leads to the production of neutralizing antibodies that enter the luminal compartment of the male reproductive tract [160]. Several antigens have been named as contenders for antispermatozoa vaccines, for which antibodies were isolated from infertile couples. A number of antispermatozoa vaccines are being successfully used to reduce the fertility rate of animals [161].

**Figure 3:**
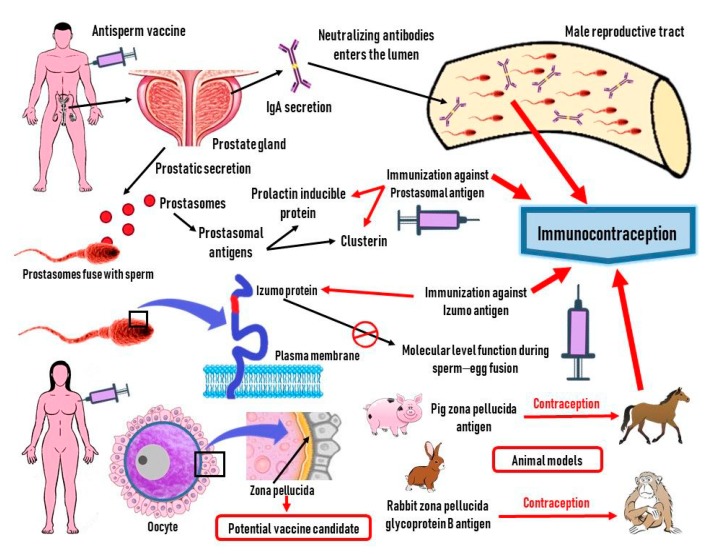
Mechanism of action that can produce successful immunocontraception along with potential antigens that can be used as vaccine candidates are represented.

Stages during the successful contraception in a female after immunization with an anti-fertility vaccine are depicted in Figure 3.

### 4.2. ASAs in Contraception

#### Sperm and Oocyte Proteome and Immunological Infertility

ASAs present in the serum samples of both infertile women and men could be used to identify immunodominant sperm antigens. Shetty et al. identified such antigens from sperm surfaces that could have a possible role in infertility and could be used as a contraceptive. Serum samples from infertile men and women containing ASAs detected with IBT and serum samples of fertile men and women without ASA detection were used for a comparative study. By using the advancements in the field of proteomics [162], it was very easy to sort out the sperm auto- and iso-antigenic protein spots by using high resolution two-dimensional (2D) electrophoresis followed by mass spectroscopy and Western blotting. Even with more advancement like vectorial labelling, a subset of six auto and iso antigens were identified with some relevance to antibody-mediated infertility [162].

ASAs as a vaccine needs to be at leastas effective than existing contraceptives in its properties and should be reversible [163,164,165,166,167,168,169]. The first attempt to evaluate the feasibility of using ASAs as a contraceptive in humans was made by Baskin et al. who intramuscularly immunized a group of 20 fertile women with approximately 3 mL of freshly ejaculated semen [170]. Three immunizations were performed in the absence of an adjuvant. One woman in this group who was immunized with a very low dose of semen produced higher levels of spermatotoxins and was infertile for over a year [170]. This type of infertility is reversible and at any time after 1 year from immunization, women may become pregnant. This method of contraception has no side effects except for a little pain at the injection site for 24 to 48 h. Many researchers who have attempted to prepare contraceptive vaccines that react with sperm have focused on a particular candidate protein. Several antisperm antibody-based vaccines has been reported after this first study’s promising results [171].

Another sperm protein called Izumo, which is located in the sperm plasma membrane, has been identified as a very good target antigen for developing immunocontraceptive vaccine for females since the protein functions at a molecular level during sperm–ovum fusion [172]. Immunizing the Izumo antigen in a mouse model exhibits inhibition in sperm–ovum interactions. Inoue et al. studied whether human Izumo is also involved in sperm and oocyte interactions during fusion [173,174]. Inoue conducted research by producing anti-human-Izumo (polyclonal antibody) and then mixing this with the incubation mixture for 45 min (sperm and ovum). This resulted in no fusion between sperm and ovum because of the inhibition by Izumo-antibodies. However, in the case of a sperm treatment with standard IgG, the fusion clearly happens. On average, six sperm were able to bind with an ovum using this in vitro experiment. Membrane cofactor protein (CD46) and isoantigenic sperm acrosomal membrane-associated protein (SAMP32) [175] were also found on the sperm surface plasma membrane. Out of all the antigens on the sperm cell plasma membrane, Izumo was found to have a significant role in fusion and it can be used as an immunocontraceptive, as evident from various in vitro and in vivo studies [176,177].

ASA targets not only sperm surface proteins but also those on the surface of prostasomes (a kind of extracellular vesicles), which may eventually add to the complexity in immuno-infertile patients [178]. Prolactin inducible protein and clusterin are found to be the most abundant target prostasomal antigens for antisperm antibodies in most immuno-infertile men [179], providing further complexity during the treatment process. These can be used as immunocontraceptive agents to control the fertility status and prevent pregnancy [178]. Prostasomes may also directly modulate the female immune reactions towards spermatozoa by getting coated over the surface [180].

The most successful antigen isolated was hyaluronidase (PH-20) which completely abolished fertility in male guinea pigs with minor side effects [181]. Fertilization antigen 1 (FA1), expressed in post capacitation, reduced female fertility rates by 70% in B6 mice [182,183,184,185]. Inhibin, a human seminal plasma protein of prostatic origin, caused infertility in 75% of male rats [186]. Sperm protein 17 (SP-17), a cytoplasmic protein that is expressed during acrosome reactions, was synthesized as an antispermatozoa vaccine and decreased fertility by 72% [187]. Another potential target for an antisperm antibody-based vaccine was sperm agglutination antigen-1 (SAGA-1), which was named as an important target in the World Health Organization antifertility vaccine studies [188,189]. CD52 antibodies, reacting with the sperm isoform, have certain limitations compared to other CD52 isoforms. S19 is a monoclonal antibody of isotype CD52 and a potential modern contraceptive vaccine [190,191,192,193]. Naz et al. identified a sperm protein and isolated only 12 amino acids of peptide YLP 12 (novel dodecamer peptide sequence). Immunizing against antigen YLP 12 was found to reduce fertility [194]. Particularly this vaccine may be rendered as a recombinant DNA-based vaccine to reduce fertility over a prolonged period in mouse models. Lactate dehydrogenase C4 is a sperm-specific antigen that may be used as an effective contraceptive in many primates [195,196,197,198,199,200,201,202,203,204]. This is formulated as a DNA-based vaccine and used to control many animal populations where applicable.

### 4.3. Oocyte Antigens in Contraception

Potential antigen candidates are mainly isolated from the zona pellucida (ZP), which is the layer surrounding the oocyte [168]. Several studies have used ZP-based immunocontracepives and antioocyte vaccines in animal models [205]. PZP (pig zona pellucida)-derived contraception was used in horses and found to work in 70% of cases [206]. SpayVac is one among the proposed porcine-derived ZP antigens used in horses for contraception and it was found that it targets mainly the ZP3 protein with a high serum immunoreactivity [207]. The rabbit homolog of zona pellucida glycoprotein B (ZPB), derived from oocyte-based antifertility, was used in monkeys and elicited antibodies that blocked fertilization capacity [208,209,210]. ZPB, with squalene and arlacel A as adjuvants, was used as an antifertility agent in baboons and was able to achieve the intended objective [211]. A DNA vaccine construct in a canine model using canine ZP (CZP3) antigen that targets DEC-205 was found to be effective in raising antibodies against ZP in both serum and vaginal secretions, suggesting its applicability for an effective contraception to control animal populations [212]. It is evident that not only sperm-related antigens, but also components isolated from any part of male and female reproductive tracts or the zona pellucida can also be used as specific targets for producing contraceptive vaccines. While undergoing such anti-zona pellucida and other oocyte-targeted contraceptive measures, the ovarian function needs to be ensured at the stages for which extrapolation of other analytical tools such as anti-mullerian hormone can be adopted [213].

### 4.4. ASA-Derived Infertility and Their Treatment

It is easy to detect or diagnose ASA levels in serum or semen, as well as circulating ASA levels, via simple techniques such as IBT assays [214]. A simple method for treating antibody-related infertility is to first clear antibodies from semen via washing or simple proteolysis [215,216]. Various immunosuppressive therapies [217] are employed by researchers to treat different forms of immune-based infertility in both men and women [218]. An in vivo study in murine models with autoimmune infertility reported the immunosuppressive effect of bone-marrow-derived mesenchymal stem cells, which was transfused intravenously, thereby suggesting its therapeutic application against autoantibodies to testicular components [219]. Much confusion exists concerning the selection of a method to treat couples with antibody-based or immunological infertility. For women with circulating ASAs or an effective immune response in the cervical mucus, assisted reproduction is the alternate method available for achieving an effective pregnancy [220,221]. In this method, selected sperm without any antibodies are injected directly into the uterus to eliminate any possibility of a sperm interacting with cervical mucus, which is the primary cause of immunologic infertility. Intracytoplamic sperm injection (ICSI) is another technique by which selected sperm is directly placed in the cytoplasm of a matured ovum. This technique mostly avoids and bypasses the effect of ASA-based issues [222,223]. The possibility of more genetic deformities being generated in ICSI/IVF-conceived pregnancies when compared to normally conceived pregnancies is still being debated [224]. Georgiou et al. confirmed that IVF/ICSI exerted certain detrimental effects on children born via this method. Another important factor is that it is the female who has to undergo treatment although the male partner is the one affected by ASA-related issues [225].

## 5. Other Methods and Modes of Contraceptives

A contraceptive is ideally required to produce a reaction blocking any vital step in the reproduction process. This may be achieved during gamete production, gamete function, or gamete outcome. In men, testosterone plays an important role in spermatogenesis at the gamete production level. Hormonal male contraception essentially involves inducing testosterone deficiency via the suppression of gonadotrophin production [226]. Hormonal contraceptives in men act by decreasing spermatogonia production and increasing apoptosis of spermatocytes and spermatids [227]. These effects can be reversed by withdrawing treatment, making hormonal contraceptives an attractive option and this is one of the most powerful advantages for using them as a contraceptive. However, weekly injections for maintaining the low serum testosterone levels are needed for contraception. This might cause adverse effects in longer run. Long-term injectablesto maintain the deficiency of testosterone were introduced to reduce the frequency of administration, but their efficiency varied in different ethnic groups. Exogenous testosterone was reported to reduce luteinizing hormone levels more in Asian men than in white men. It was also reported that the hypothalamus-luteinizing hormone axis was more sensitive in Asian men [228]. Similar reports regarding different levels of efficacy exhibited by hormonal contraceptives in various ethnic groups have been published [229]. East Asians were found to be sensitive to the use of testosterone alone as a contraceptive, whereas Caucasians required additional agents, such as progestin, to provide a comparable level of contraceptive protection.

To produce a hormonal contraceptive effective for all human male populations, combinations of testosterone and etonogestrel, norethisterone, and medroxyprogesterone were used [230]. More research on enhancing the effects of hormonal contraceptives is ongoing. These include delivering higher doses of androgens through skin patches and identifying new non-traditional androgen agonists [229]. Appropriate focus needs to be given regarding interfering with highly specific aspects of spermatogenesis, such as particular enzymatic processes and intercellular talks via cytokines, or exploiting antibodies against antigens of the epididymis or the spermatozoa. Better knowledge of normal and pathological spermatogenesis will facilitate development of effective male contraceptives [231].

Usage of human chorionic gonadotropin (hCG) to arrest pregnancy during the gamete outcome stage is a contraceptive option. Reversible fertility control, via the usage of the beta subunit of hCG covalently bound to gonadotropin, has been reported in females [231]. This vaccine was reported to be safe with no side effects in phase 1 trials. However, Stephen et al. has warned against undesirable effects that hCG may induce by stimulating antibodies that apparently increase the bioavailability of hCG. They further reported on the presence of hCG and its receptor-like proteins in non-pregnant women. These observations led to a conclusion favoring the interruption of gamete function as the ideal target for contraception as this may prevent fertilization without interrupting the potential for pregnancy [231].

## 6. Patents Related to ASAs and Their Application as Contraceptives

Immunocontraception offers great advantages over other techniques. It does not require surgical intervention and does not require daily administration. It is devoid of the side effects associated with hormonal contraceptives as well. Encouraged by the array of advantages associated with the use of immunocontraceptives, many researchers have patented viable products targeting sperm, ova, or embryos, which prevent fertilization.

Immunocontraceptive products conceptualized and patented using different native or allo proteins related to reproductive functions are summarized in Table 1.

## 7. Concluding Remarks and Future Perspectives

Sperm immunology provides useful information for clinicians. Seminal ASA levels were found not only in infertile cases, but also in fertile men and women. These findings reveal that a low concentration of ASAs is found even in fertile men. Intrauterine insemination (IUI), ICSI, and IVF were found to be the treatments most suited for severe immunologic infertility and infertility due to unknown causes as there is no alternative. These findings were the results of detailed and systematic meta-analyses indicating that seminal ASAs do not directly hamper pregnancy rates through IVF or ICSI. This suggests that these assisted reproductive techniques may be a viable option for infertile couples. However, even more sophisticated studies are warranted to address issues such as determination of ASA threshold levels, among others. Circulating ASAs have been proven to play a partial role in missed abortions during pregnancy. This review also concludes that ASAs may play a negative role in sperm motility, sperm production, sperm transport, acrosome reaction, and capacitation. Certain inconsistencies related to the impact of ASAs on male and female reproductive efficiency or outcomes still exist, reflecting inadequacies associated with prevailing diagnostic techniques. Thorough antigenic characterization of ASAs from each individual male and female subject is needed to locate target antigens within sperm, as well as the fertilization stage at which each type of ASA interferes. These forms of vaccines are non-invasive, and are more effective and cheaper compared to existing contraceptive methods, which require a lot of effort to be used in humans.

Recently, promising research studies focusing on different aspects of ASAs in the reproductive biology of humans and other species have been considered. The usefulness of sperm antigens or ASAs (recombinant) as human/animal contraceptive vaccines has been revealed but is yet to be validated via clinical testing. Progress has been made in research associated with the development of techniques to overcome ASA-induced infertility in people who wish to have children. The process of ASA generation in both human males and females is poorly understood and is a subject area that deserves further attention and research. Various arguments have been put forward regarding the testing of males for ASAs on a routine basis, but it is assumed that such approaches are not always mandatory. Therefore, all the above findings show that ASAs impair the motility of spermatozoa, transport of sperm through the female reproductive tract, sperm survival, acrosomal reactions, fusion of sperm, and oocyte and embryo development. Since specific targeted therapies against ASAs are not in practice yet, assisted reproductive technologies are the appropriate alternative choices for healthy conception.

## Figures and Tables

**Table 1:** Patents related to antisperm antibodies and male contraception.

Title of the Patent	Patent Number	Details on the Invention	Inventors	Reference
Sperm antigen corresponding to a sperm autoantigenic epitope and methods of using the same	US5175148	Usage of rabbit sperm membrane autoantigen for detecting auto immune infertility and as an immune contraceptive agent.	Michael G. O’Rand, Esther E. Widgren	O’Rand and Widgren, 1992 [232]
DNA immunocontraceptive vaccines and uses thereof	WO2005093043A1	Recombinant bacteria possessing sperm specific protein-lactate dehydrogenase C (LDH C). On administration, antibodies will be produced against LDH C leading to less sperm in males and immunocontraceptive in females.	Barrie G. Kitto, Daniel C. Hirschhorn	Kitto and Hirschhorn, 2005 [233]
Sperm antigen corresponding to a sperm zona binding protein autoantigenic epitope	US5480799 A	Human seminal plasma 17 protein and its antigenic fragments were used as an immunocontraceptive.	Michael G. O’Rand, Esther E. Widgren, Richard T. Richardson, Isabel A. Lea	O’Rand et al., 1996 [234]
Fertility control	US4879285	Platelet activating factor (PAF) is an enhancer of embryo viability. PAF antibody in conjugation with estrogen was proposed to have a contraceptive effect.	Christopher O’Neill	O’Neill, 1989 [235]
Use of mullerian inhibiting substance (MIS) as a contraceptive agent	US4753794	MIS interferes in the maturation of oocytes and thus prevents formation of the embryo.	Patricia Donahoe	Donahoe, 1988 [236]
Antigenic modification of polypeptides	US4713366A	Both endogenous and exogenous proteins are modified to become more antigenic so as to produce antibodies against host components, including sperm.	Vernon C. Stevens	Stevens, 1987 [237]
Human sperm specific lysozyme-like proteins	US7125550B2	Testis specific proteins C 19 and C 23 were modified to be used as contraceptive agents.	John C. Herr, Friederike Jayes, Arabinda Mandal, Jagathpala Shetty, Michael J. Wolkowicz	Herr et al., 2006 [238]
Compositions and methods for reducing or preventing fertilization in fish and birds	US6790457B1	Immunocontraceptive vaccine was developed using a homolog of the Zona pellucid protein along with a carrier.	Robert Brown, Bill Pohajdak, Janet Horrocks, Leslie McLaren	Brown et al., 2004 [239]
Topical application of antibodies for contraception and for prophylaxis against sexually transmitted diseases	US6355235B1	Antibody molecules capable of immobilizing sperm can be used in men as a topical application, and in women, these antibodies were made to be released using intrauterine/intravaginal devices.	Richard A. Cone, Kevin J. Whaley	Coneand Whaley, 2002 [240]
Method to prevent fertilization in mammals by administering a single dose of zona-pellucida-derived antigens, liposome, and Freund’s adjuvant	US5736141A	A liposome mediated delivery of zona pellucida antigens through vaccination was reported to produce antibodies detected for 22 months.	Robert Brown, Michael Mezei, Bill Pohajdak, Warwick Charles Kimmins	Brown et al., 1998 [241]
Chimeric contraceptive vaccines	US6045799A	Carrier protein related with a protein of reproductive function was used.	Jerry J. Reeves, Kevin P. Bertrand, Yuzhi Zhang	Reeves et al., 2000 [242]
Antigens for immunocontraception	US7056515B2	Immunocontraceptive vaccines were produced using zona pellucida protein fragments from carnivorous animals.	Robert George Brown, Marc Mansour, Bill Pohajdak	Brown et al., 2006 [243]
Rabies-virus-based recombinant immunocontraceptive compositions and methods of use	US8524247B2	Nucleic acid information of an immunocontraceptive protein in a recombinant rabies virus was proposed to inhibit fertility.	Xianfu Wu, Charles Rupprecht	Wuand Rupprecht, 2013 [244]
Methods and compositions for stable transgenic plant pharmaceuticals and their use as contraceptives	CA2441699A1	Pharmaceutical proteins capable of acting as an immunocontraceptive were proposed to be produced in transgenic plants.	Dwayne Kirk, Hugh Mason, Amanda Walmsley, Charles Arntzen	Kirk et al., 2004 [245]
Protein for the immunocastration of mammals	US8940693B2	A fusion protein for immunocastration was used in this method.	Leonardo Enrique Saenziturriaga	Saenziturriaga, 2015 [246]
Methods of inhibiting gonad Maturation	US 2015/0104474 A1	Nucleic acid molecules possessing compounds capable of eliciting immune response against gonad maturation in juvenile animals was developed	Igor Babiak, Reid Hole	Babiak and Hole, 2018 [247]
Immunogenic LHRH (synthetic luteinizing hormone releasing hormone) compositions and methods relating thereto	US8741303B2	Fertility potential was modified using LHRH with five amino acids attached to a C terminal.	Michael Kerin McNamara	McNamara, 2014 [248]
Intratesticular injection of immunogens	US 2014/0271716 A1	Chemical sterilization agent along with immunogen against disease pathogens when introduced in testis tissue can bring effective contraception.	Min Wang	Wang, 2014 [249]
Antibody-mediated immunocontraception	US20140223591A1	Methods and compositions for contraception (including vector-based approaches) and manipulation or other reproduction-associated traits.	Bruce A. Hay, Juan Li	Hay and Li, 2014 [250]
Method for the prediction of immune-dependent male infertility development in patients after varicocelectomy	UA102086U	Laboratory-based methods and standards for determining the probability of male infertility out of immune reaction in patients having undergone a varicocelectomy.	Myroslavivna H.A., Volodymyrivna, C.V., Andriiovych, N.Y., Yosypivna, K.I., Maciej, K.	Myroslavivna et al., 2015 [251]
Medicine for treating immune male infertility	CN105079573A	A polyherbal preparation against infertility by improving immune functions and eliminating hypersensitivity and autoimmune responses.	Qingdao Huaren Tech Incubator	Qingdao Huaren Tech Incubator, 2015 [252]
Pharmaceutical composition for curing immune infertility	CN105560668A	Involves traditional Chinese medicine using a polyherbal combination for curing immune infertility.	Chen Hong	Hong, 2016 [253]
Detection methods and kits of anti-ACTL7a antibody and anti-GAPDH-2 (glyceraldehyde-3-phosphate dehydrogenase) antibody, and uses of kits	CN106153938A	This involves the diagnosis of immunological infertility through the detection of antifertility antibodies against ACTL7a (Actin-like protein 7A) and GAPDH-2 using kits provided with a purified ACTL7a antigen-coated plate and/or a purified GAPDH-2-antigen-coated plate.	Fu Jun, Song Wei, Wang Linfang, Yan Shiying, Wang Yong, Zhang Xiaodong, Yao Rongyan, Luo Yanyun.	Jun et al., 2016 [254]
*Albizzia julibrissin* and *Rhizoma cyperi* pill	CN107050378(A)	Involves the preparation of a watered pill prepared out of 19 Chinese traditional medicines with a high efficiency in treating positive antisperm antibodies.	Cui Yue	Yue, 2017 [255]
Placental chondroitin sulphate an immunogenic composition and application	WO2019075893A1	A contraceptive drug formulation for humans and other animals using placental chondroitin sulphate A to prevent implantation of the embryo onto the endometrium. Also supplied with an immunopotentiator thatcan terminatepregnancy.	Zhang Juzuo, Fan Xiujun, Zhang Jian, Chen Zhilong, Wang Baozhen, Huang Chen, Chen Jie, Li Mengxia	Juzuo, 2019 [256]
Multi-functional beverage containing peptides	CN109363037 (A)	This is a novel formulation of peptides from various sources with multiple health benefits to male subjects including immune boosting and being effective against infertility.	Wang Jinming	Jinming, 2019 [257]
Macro web bombinator antisperm polypeptide and its preparation process and use in medicine	CN1142182C	A polypeptide prepared out of novel process possessing sperm-brake activity and the peptide can be therapeutically applicable for contraception and against pancreatitis.	Yun Zhang, Zhangyun, Lai Wei, Zheng Yongtang, Li Wenhui	Zhang et al., 2004 [258]
Biological female contraceptives	ES2671345T3	Commensal *Lactobacillus* bacterium of the female genital tract has been genetically modified to express antibodies with an anti-sperm property so as to be used as an effective biological anti-fertilizing agent.	Rachel Teitelbaum	Teitelbaum, 2018 [259]
Biologic female contraceptives	JP2019017389A	Commensal microbial organisms genetically engineered to carry fragments of anti-sperm activity and applied for preventing fertilization.	Teitelbaum Rachel	Rachel, 2019 [260]
Biologic female contraceptives	KR101927747B1	Symbiotic organism genetically modified to provide effective contraception in females.	Rachel Tetelbaum	Tetelbaum, 2013 [261]
Male contraceptive compositions and methods of use	JP6022442B2	It describes the novel methods and compositions for developing compounds based on inhibitors of the bromodomain testis-specific protein (BRDt) for effective contraception in male subjects.	Bradner, James Elliott, Martin Matzuk, Jun Qi	Bradner et al., 2016 [262]
CRISP (Cysteine-rich secretory protein) polypeptides as contraceptives and inhibitors of sperm capacitation	US7405202B2	It describes the usage of a CRISP polypeptide for contraception through inhibiting fertilization events in sperm such as capacitation and acrosomal reactions.	David W. Hamilton, Kenneth P. Roberts, Kathy M. Ensrud	Hamilton et al., 2008 [263]
